# Aortic arch obstruction neonates with biventricular physiology: left-open compared to closed inter-atrial communication during primary repair – a retrospective study

**DOI:** 10.1186/s13019-015-0258-1

**Published:** 2015-04-17

**Authors:** André Rüffer, Caroline Bechtold, Ariawan Purbojo, Okan Toka, Martin Glöckler, Sven Dittrich, Robert Anton Cesnjevar

**Affiliations:** 1 Department of Pediatric Cardiac Surgery, University Hospital Erlangen, Friedrich-Alexander-University Erlangen-Nürnberg (FAU), Loschgestraße 15, 91054 Erlangen, Germany; 2 Department of Pediatric Cardiology, Friedrich-Alexander-University Erlangen-Nürnberg (FAU), Germany, University Hospital Erlangen, Loschgestraße 15, Erlangen, 91054 Germany

**Keywords:** Aortic arch reconstruction, Congenital, Neonates, Primary repair, Fenestration, Atrial septal defect, Inter-atrial communication, Hypoplastic left heart, Borderline

## Abstract

**Background:**

Leaving an inter-atrial communication (IAC) open for left atrial decompression is often recommended in neonates with aortic arch obstruction undergoing primary repair. In this study, outcomes in these patients were compared to those with intact atrial septum after repair.

**Methods:**

Between 2000 and 2013, 53 consecutive neonates with severe aortic arch obstruction (hypoplasia: n = 45, interruption: n = 8) underwent primary repair from an anterior approach. Median age and weight were 8 days (range: 2–30) and 3.2 kg (range: 2.4-4.4), respectively. Cardiac morphology included a ventricular septal defect (VSD, large: n = 28, small: n = 7), malposition of great arteries (n = 10), and severe left ventricular outflow tract obstruction (LVOTO, n = 10). During corrective surgery IAC was closed (group-I, n = 37) or partially left-open (group-II, n = 16). Primary endpoints were hospital death, and re-intervention (surgery and/or balloon) due to aortic arch re-coarctation or recurrent LVOTO. Statistically significant variables by univariate analysis were incorporated in the corresponding multivariable regression model.

**Results:**

Regarding morphological discrepancies more patients in group-II presented with LVOTO (p = 0.05), or the combination of arch hypoplasia, intact ventricular septum and normal ventriculo-arterial connection (p = 0.017). Hospital mortality was 8.1% in group-I and 37.5% in group-II (p = 0.016). Re-intervention was performed in 13 patients (group-I: n = 6 vs. group-II: n = 7) due to aortic arch re-coarctation (n = 12) and/or recurrent LVOTO (n = 3), and resulted in a Kaplan-Meier freedom from re-intervention of 87 ± 6% and 79 ± 8% in group-I, and 64 ± 14% and 64 ± 14% in group-II after 1 and 5 years, respectively (p = 0.016). Multivariate analysis revealed LVOTO as an independent risk factor for hospital death (p = 0.042), whereas both LVOTO and left-open IAC (p = 0.001 and 0.01) were independent risk factors for re-intervention.

**Conclusions:**

A left-open IAC increases risk of re-intervention at the left heart aorta complex. Sustained left-to-right shunting on atrial level seems to induce preload reduction of the often restrictive left ventricle leading to decreased aortic blood flow.

## Background

Neonates with ductal dependent systemic circulation of the descending aorta require surgery or intervention during the first weeks of life. Antegrade blood flow through the ascending aorta and the branches of the aortic arch is an important condition indicating biventricular physiology in patients with severe aortic arch obstruction [1]. Primary repair from an anterior approach is achieved by reconstruction of the aortic arch and simultaneous correction of concomitant cardiac malformations, if present [2-7].

The heterogeneity of aortic arch obstructions, ventriculo-arterial connections, and intra-cardiac abnormalities requires an individual consideration for each patient. Left ventricular morphology can vary between an intact ventricular septum with sometimes a small muscular ventricular septal defect (VSD), and a large, non-restrictive VSD. In presence of preserved right ventricular structures and a pressure equalizing VSD, the left ventricle is usually well-developed and a biventricular repair should be achievable [1,8]. On the other hand, an intact ventricular septum is often associated with hypoplasia of all structures of the left heart-aorta complex with or without intrinsic valve stenosis or atresia resembling the hypoplastic left heart syndrome, where a Norwood procedure is preferred [8].

Hypoplastic but morphologic normal left ventricle is a clinical entity which remains difficult to define [9]. The peculiar question of biventricular repair or rather univentricular pathway is answered by echocardiographic measurements or surgeon’s experience, and has led to different and sometimes contradictory approaches regarding corrective surgery protocols. Particularly by achieving biventricular repair, general agreement does not exist regarding the management of the frequently associated open inter-atrial communication (IAC) [10]. Complete elimination of the left-to-right shunt by closing the atrial septal defect (ASD) has been suggested to reduce the risk of low cardiac output due to excessive left-to-right shunt through an unrestrictive communication. Improvement of left ventricular filling and therefore subsequent left ventricular growth is expected [1,10,11]. On the other hand, the sudden increase of preload may result in left ventricular overload and distension determining severe left ventricular dysfunction with sometimes a “stormy” early postoperative period [1,9,10]. An alternative surgical option as a compromise between both approaches is the partial ASD-closure with a fenestrated patch [10,11].

The goal of this study was to identify predictors of survival and cardiac re-intervention for neonates with aortic arch obstruction undergoing primary repair. Hereby, the role of a left-open IAC was elucidated.

## Methods

### Study design

The study was notified to the local ethics committee. The records of all neonates (age < 30 days) with aortic arch obstruction, antegrade flow across the ascending aorta and ductal dependent lower body perfusion, who underwent primary repair from an anterior approach between January 2000 and December 2012, were retrospectively reviewed. Demographic, anatomic, and perioperative data were retrieved from the patient’s medical record.

Uni-and multivariate risk analysis for the whole cohort was calculated for mortality and the need for surgical or catheter based cardiac re-intervention.

Regarding management of the inter-atrial septum, patients were divided into 2 groups (group I, n = 37: IAC-closed, and group II, n = 16: IAC-open). Group related outcome was evaluated with respect to early and long-term survival, and re-intervention at the left heart aorta complex.

### Patient characteristics

Fifty-three consecutive neonates with hypoplastic (n = 45) or interrupted aortic arch (n = 8, all type B) were included into the study. Median age at surgery was 8 days (range, 2–30) and median weight 3.2 kg (range, 2.4–4.4), 37 patients (70%) were male. A large (non-restrictive) VSD was present in 28 patients (53%), in 7 patients (13%) VSDs were considered small (restrictive). One patient with arch interruption had an aorto-pulmonary window and was counted to patients with a large VSD. Ten patients (19%) had malposition of great arteries, including D-transpositon (D-TGA; n = 7), Taussig-Bing anomaly (n = 2) and congenitally corrected transposition (n = 1). A significant left ventricular outflow tract obstruction (LVOTO) was defined as a diameter smaller than 5 mm or a Z-score < −2, measured by echocardiography at aortic subvalvar or valvular level, and was present in 10 patients (19%).

### Surgical technique

The surgical technique of aortic arch repair has been previously described by our group [12]. The majority of patients was operated with antegrade cerebral low-flow perfusion (n = 43, 81%), or alternatively by using deep hypothermic circulatory arrest (n = 16, 30%), 6 patients had a combination of both methods. In 21 patients (40%), aortic arch repair was performed on the beating heart with selective myocardial perfusion. Myocardial protection for the remaining patients and for all intra-cardiac procedures was achieved by cold crystalloid cardioplegia (30 ml/kg; Custodiol©, Köhler Chemie, 64625 Bensheim, Germany). Aortic arch reconstruction technique included excision of the coarctation with dorsal end-to-end aortic re-anastomosis and anterior patch augmentation in all except 1 patient, where direct end-to-side anastomosis of the descending with the ascending aorta was performed. Intra-cardiac repair included closure of a large VSD (n = 28) with a patch using a continuous or interrupted suture, as appropriate. Small muscular VSDs (n = 7) were not addressed in favour of a later spontaneous closure. ASD-closure (group-I) was performed either directly or with a patch. Two patients with intact atrial septum were part of group-I. A left-open IAC (group II) was achieved either by partial closure of a secundum ASD with a direct suture (n = 4), central fenestration of an ASD-patch with a 4 mm punch (n = 1), leaving a non-intentional residual ASD (n = 1), ASD-enlargement (n = 2), or by not addressing the PFO (n = 8). Nine patients (17%) underwent the arterial switch operation. The aortic valve had to be addressed by commissurotomy in 3 patients. Ross-Konno and Yasui procedure were performed in 1 patient each, respectively.

### Statistics

Cardiac surgical procedures were categorised according to the Aristotle basic and comprehensive scores. Death was defined as early death in the first 30 days after primary repair, hospital death as death occurring during hospital stay after primary repair. Follow-up was complete, with a mean duration of 6.0 ± 3.8 years (median: 5.6 years; range, 53 days–14 years) in December 2013 and was accomplished by routine control echocardiography in our hospital or by direct contact with the referring cardiologist. Re-intervention was defined as secondary surgery or balloon (± stent) for aortic arch re-coarctation, and/or recurrent LVOTO.

Descriptive data are presented as mean (± standard deviation) or median (range) for continuous variables, and frequencies and percentages for categorical variables. Statistical group difference and univariate risk factors for hospital death were calculated by using Fisher’s exact test or *X*^2^ test for categorical variables, and *T*-test or Mann–Whitney-test for continuous variables, as appropriate. Freedom from re-intervention was estimated according to the Kaplan–Meier method. Univariate potential predictive factors of re-intervention were identified using the log-rank test for categorical variables and Cox-regression for continuous variables. Included numeric variables were age, preoperative weight, and, duration of CPB, aortic cross-clamping, antegrade cerebral perfusion, and deep hypothermic circulatory arrest, respectively. Included categorical variables (yes/no) regarding morphological characteristics were aortic arch anatomy (hypoplasia or interruption), VSD (large or small, or intact ventricular septum), ventriculo-arterial connection (normal or malposition of great arteries: TGA, Taussig-Bing anomaly), LVOTO. Variables regarding organ protective techniques were selective myocardial perfusion, antegrade cerebral perfusion, and deep hypothermic circulatory arrest; variables regarding aortic arch repair techniques included different patch material (autologous or bovine pericardium, homograft-patch). Univariate variables with P-value of < 0.10 were incorporated in the multivariable analysis which was performed by stepwise logistic regression for hospital death, and Cox proportional hazard analysis for re-intervention, respectively. Results of the logistic regression model were presented as odds ratios (ORs) with 95% confidence intervals (95% CI), and of the Cox proportional hazard analysis as hazard ratios (HRs) with 95% CI. Statistical group difference and univariate analysis was calculated using SPSS version 21.0 (SPSS, Inc., Chicago, IL, USA). Multivariate analyses were conducted using R version 3.0.3 (R Project for Statistical Computing, Vienna, Austria). A P-value of < 0.05 was considered statistically significant.

## Results

### Patient characteristics and operative data

Group related data regarding patient characteristic, anatomic varieties and operative techniques are represented in Table 1. More patients in group-II presented with LVOTO (p = 0.05) and/or the combination of aortic arch hypoplasia, intact ventricular septum and normal ventriculo-arterial connection (p = 0.017). Accordingly, VSD-patch closure was more prevalent in group-I (p = 0.038).

**Table 1:** **Group differences: patient characteristics and operative data**

	Group-I (IAC-closed)		Group-II (IAC-open)		
	N	% or Mean ± SD	N	% or Mean ± SD	p
Aristotle basic score	37	11.0 ± 2.5	16	11.0 ± 3.5	0.92
Aristotle comprehensive score	37	13.4 ± 4.1	16	14.3 ± 4.9	0.46
Age (days)	37	8.6 ± 4.4	16	9.6 ± 6.6	0.59
Weight (kg)	37	3.2 ± 0.5	16	3.2 ± 0.5	0.75
Gender-male	29	78	8	50	0.054
Aortic arch hypoplasia	30	81	15	93	0.41
Aortic arch interruption	7	19	1	6	0.41
VSD (all)	27	73	8	50	0.11
VSD (large)	23	62	5	31	0.038
Intact ventricular septum	14	38	11	69	0.038
Transposition of great arteries	8	22	2	13	0.70
- Taussig-Bing	1	3	1	6	0.52
- D-Transposition	6	16	1	6	0.66
- L-Transposition	1	3	0	0	1
LVOTO	4	11	6	38	0.050
Selective myocardial perfusion	16	43	5	31	0.41
(min)	37	19 ± 22	16	13 ± 20	0.49
Antegrade cerebral perfusion	30	81	13	81	1
(min)	37	33 ± 29	16	35 ± 31	0.85
DHCA	11	30	5	31	1
(min)	37	7 ± 12	16	6 ± 8	0.59
CPB (min)	37	177 ± 66	16	213 ± 147	0.88
X-clamp (min)	37	65 ± 38	16	80 ± 74	0.70
Arterial switch operation	7	19	2	13	0.66
VSD-closure	23	62	5	31	0.038
Aortic valve repair	1	3	2	13	0.21

CPB: cardiopulmonary bypass; DHCA: deep hypothermic circulatory arrest; LVOTO: left ventricular outflow tract obstruction; SD: standard deviation; VSD: ventricular septal defect X-clamp: aortic cross-clamp.

### Survival

**Figure 1:**
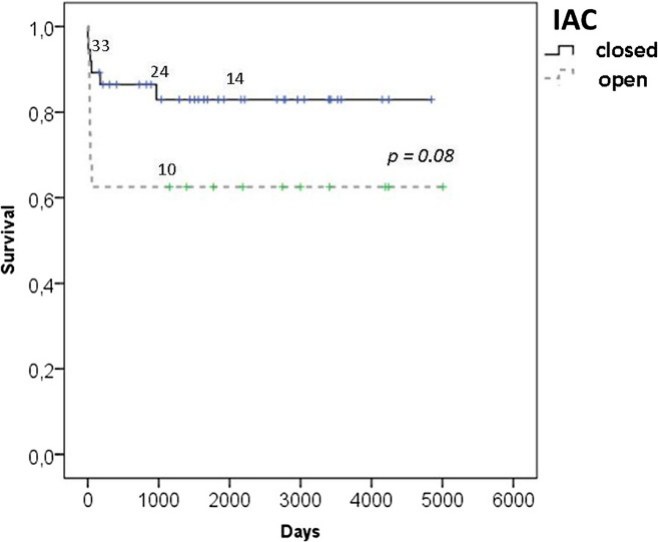
Kaplan Meier survival stratified by IAC-closed and IAC-open.

Early mortality was 5.4% in group-I and 25.0% in group-II (p = 0.060). Hospital mortality was 8.1% in group-I and 37.5% in group-II (p = 0.016). Kaplan-Meier survival after 1 and 5 years was 86 ± 6% and 83 ± 6% in group-I, and 63 ± 12% and 63 ± 12% in group-II, respectively (p = 0.082) Figure 1.

Cardiac anatomy and surgical procedures of patients who died in hospital are represented in Table 2.

**Table 2:** **Hospital death and/or re-intervention**

Year	Diagnosis	Concomitant procedure	Further surgery/intervention	Death
Group-I (IAC-closed)				
2001	IAA-VSD	VSD-patch	no	hospital death
	PFO	PFO-closure		day 1
2004	HAA-VSD	VSD-patch	Re-CoA	alive
	PFO	ASD-closure	surgery, day 144	
2004	HAA-IVS	ASD-closure	Re-CoA	alive
	PFO		surgery, day 556	
2006	HAA-IVS	ASD-closure	Re-CoA	alive
	LVOTO		surgery, day 276	
	PFO			
2007	HAA-VSD	VSD-patch	Re-CoA, Re-LVOTO:	alive
	PFO	ASD-closure	surgery, day 897	
2007	HAA-VSD	ASD-closure	no	hospital death
	ASD-II			day 35
2010	HAA-VSD	VSD-patch	Re-CoA, Re-LVOTO:	(late) death
	LVOTO	AVR	balloon, day 144	day 176
	ASD-II	ASD-closure	Ross-Konno, day 156	
2010	HAA-IVS	Arterial switch	no	hospital death
	D-TGA	ASD-closure		day 7
	ASD-II			
2013	IAA-VSD	VSD-patch	Re-CoA	alive
	D-TGA	ASD-closure	surgery (day 217)	
	ASD-II			
Group-II (IAC-open)				
2000	HAA-IVS	PFO left-open	Re-CoA	alive
	PFO		balloon, day 3950	
2001	HAA-IVS	AVR	(Norwood-I	hospital death
	LVOTO	ASD-enlargement	day 28)	day 29
	PFO			
2002	IAA-VSD	VSD-patch	Re-CoA	alive
	ASD-II	partial ASD-closure	balloon, day 2812	
2004	HAA-IVS	PFO left-open	Re-Co-A	alive
	PFO		balloon, day 2191	
2005	HAA-IVS	ASD-fenestration	(ASD-closure	hospital death
	LVOTO		day 22)	day 25
	ASD-II			
2006	HyAA-IVS	no	Re-CoA	alive
			surgery, day 213	
2007	HAA-VSD	Arterial switch	(ASD-closure	hospital death
	D-TGA	VSD-Patch	day 4)	day 14
	ASD-II	partial ASD-closure		
2007	HAA-VSD	VSD-patch	Re-CoA	alive
	PFO	ASD left-open	surgery,.day 73	
2008	HAA-IVS	ASD left-open	(Norwood-I	hospital death
	LVOTO		day 16)	(day 37)
	ASD-II			
2009	HAA-IVS	AVR	Re-LVOTO	alive
	LVOTO	ASD-enlargement	Ross-Konno, day 24	
	PFO			
2009	HAA-IVS	Ross-Konno,	Re-Co-A	hospital death
	LVOTO	PFO left-open	balloon, day 36	(day 56)
	PFO			
2010	HAA-VSD	Ross-Konno,	no	hospital death
	LVOTO	PFO left-open		(day22)
	ASD-II	ASD-enlargement		

Re-intervention was defined as surgery or balloon for aortic re-coarctation or recurrent LVOTO. Concomitant procedure was defined as other procedure than aortic arch reconstruction during primary repair. ASD: atrial septal defect: AVR: aortic valve repair (commissurotomy); HAA: hypoplastic aortic arch; IAA: interrupted aortic arch; IAC: inter-atrial communication; IVS: intact ventricular septum; LCO: low cardiac output; LVOTO: left ventricular outflow tract obstruction; PFO: persistent foramen ovale; Re-CoA: aortic re-coarctation; VSD: ventricular septal defect.

### Re-intervention

**Figure 2:**
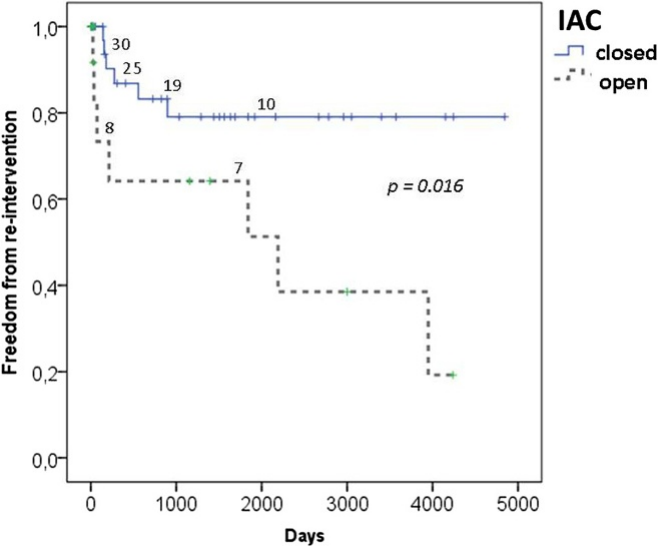
Kaplan Meier freedom from re-intervention stratified by IAC-closed and IAC-open.

Thirteen patients (group-I: n = 6 vs. group-II: n = 7; log-rank p = 0.016) required 8 surgical and 5 catheter based re-interventions due to aortic arch re-coarctation (n = 12), or recurrent LVOTO (n = 3), respectively; 2 patients had both procedures. Kaplan-Meier freedom from re-intervention was 87 ± 6% and 79 ± 8% in group-I, and 64 ± 14% and 64 ± 14% in group-II after 1 and 5 years (p = 0.016), respectively Figure 2.

Cardiac anatomy and surgical procedures of patients who had re-intervention are represented in Table 2.

### Risk analysis

Univariate risk analysis showed that LVOTO (p = 0.008), CPB-time (p = 0.026), and left-open IAC (p = 0.016) were risk factors for hospital death. Logistic regression revealed LVOTO as the only independent risk factor for hospital death (p = 0.042) Table 3.

**Table 3:** **Multivariate analysis: logistic regression for hospital mortality**

	OR	95% CI	p-value
LVOTO	6.3	1.05 – 39.65	0.042
CPB-time	1.004	0.99 – 1.01	0.24
IAC-open	3.9	0.7 – 24.1	0.13

CI: confidence interval; CPB: cardiopulmonary bypass; IAC: inter-atrial communication: LVOTO: left ventricular outflow tract obstruction; OD: odds ratio.

Significant univariate risk factors predicting re-intervention were LVOTO (p = 0.001), and left-open IAC (p = 0.016), and were included for multivariate analysis. Cox proportional hazard analysis revealed LVOTO, and left-open IAC as independent risk factors for re-intervention (p = 0.001 and 0.01, respectively) Table 4.

**Table 4:** **Multivariate analysis: cox regression for re-intervention**

	HR	95% CI	p-value
LVOTO	11.1	2.6 – 48.4	0.001
IAC-open	5.2	1.5 – 18.3	0.01

CI: confidence interval; HR: hazard ratio; IAC: inter-atrial communication: LVOTO: left ventricular outflow tract obstruction.

## Discussion

Blood flow is the main stimulus for growth of cardiovascular components. Any structural or volume related lesion which impairs blood flow through the left ventricle would be expected to lead to underdevelopment of downstream structures [9,11]. LVOTO is a structural barrier which is associated with left ventricular hypertrophy and reduced left ventricular end-diastolic volume. On the other hand, an ASD decreases preload of the left ventricle by draining blood from the left atrium into pulmonary circulation. In this study, outcome of neonates with aortic arch obstruction who underwent primary repair was evaluated.

This study shows that in neonates with aortic arch obstruction and biventricular physiology, a preexisting LVOTO or left-open IAC during primary repair were both independent risk factors for re-intervention at the left ventricular outflow tract-aorta complex. Re-intervention was 18 times more probable in patients where the inter-atrial septum was left-open than closed. We hypothesize that sustained left-to-right shunting on atrial level might cause preload reduction of the often borderline left ventricle leading to decreased blood flow across the left heart and less growth of left ventricular outflow structures [9,11].

It has been previously demonstrated, that re-establishment of a near normal preload to the left ventricle restores left ventricular output and size [9,13,14]. In patients with hypoplastic left heart complex, Tchervenkov et al. recommended complete relief of the systemic obstruction and elimination of all intra-cardiac shunts to fully preload the heart, enabling growth of the left ventricle after biventricular type of repair [1,8]. Daebritz et al. described a series of successful biventricular correction of patients with aortic coarctation and emphasized the need of closing all intra-cardiac shunts to force the entire cardiac output through the left heart to give the impulse for adequate growth [11].

On the other hand, Serraf et al. reported their experience regarding coarctation repair in ductal-dependent neonates with hypoplastic but morphologically normal left ventricle, and recommended, that normalization of loading conditions should not be performed in a single stage operation. Forcing the entire output through the left ventricle by closure of any atrial shunt would result in left ventricular overload and distension [9]. The authors hypothesized, that “since the neonatal left ventricle is already functioning at the plateau of the Starling’s curve, any volume overload can determine severe left ventricular dysfunction, and eventually death”. We think this is a very ambivalent point. The dogma of Frank-Starling implies that “the heart will pump what it receives” which means, that stroke volume is increased by a rising preload. When the plateau of Starling’s curve is reached further dilatation of the left ventricle cannot be answered by increased output, leading to left ventricular deterioration. However, in neonates where prostaglandins are used to enable distal aortic perfusion, the right heart has to support both the pulmonary and part of the systemic circulation, as it has being used during fetal circulation. Following birth and shifting from fetal to neonatal circulation, the left ventricular muscle without endocardial fibroelastosis is able to stretch relative to the increase of cardiac output demanded for additional lower body perfusion [9]. Former studies evaluating left ventricular size in patients with aortic stenosis, have suggested that a minimal end-diastolic left ventricular volume of 20 mL/m^2^ of body surface area enables a biventricular repair [15,16]. For patients without intrinsic valve stenosis much smaller ventricles, even in neonates with left ventricular end diastolic volume of 10 mL/m2 and non-apex forming left ventricle, significant growth of the left ventricular dimension has been proven [9-11]. Therefore we would doubt that the plateau of the Starlings curve is always reached, neither in newborn patients, nor in most patients with HLHC, and we would suggest that even a complete closure of all intra-cardiac shunts should be answered by increased cardiac output.

However, following biventricular repair in patients with left heart hypoplasia, complete closure of the inter-atrial communication has been reported with a “stormy” postoperative course, due to elevated left atrial pressure which usually normalizes after 24–48 hours [1,10]. As a compromise, in order to protect the heart from acute overload and distention, Tchervenkov and colleagues changed their strategy to creating a 2- to 3-mm inter-atrial fenestration allowing a stepwise adaptation of the left heart structures to the new loading conditions [1]. In our study cohort closure of the ASD did not result in higher mortality and we suppose that patients who died with a closed ASD would have died even with a left-open ASD. However, all left-open IAC were greater than 4 mm in diameter. Perhaps ASD-closure with a patch using a 2-3 mm fenestration as recommended by the group mentioned above would have achieved a different outcome [1,10].

Nevertheless, we doubt the benefit of left atrial decompression by a left-open IAC during primary repair of aortic arch obstructive lesions. Our strategy is to close all intra-cardiac shunts, if possible. Nowadays new pharmacological options in optimizing not only systolic function but even diastolic compliance of a borderline left ventricle undergoing complete repair have been introduced by the preoperative administration of calciumsensitizer [17,18]. It has become standard practice in our hospital to load neonates with complex single or borderline left ventricle morphology with Levosimendan before surgery. We believe that this preparation of the hypertrophied left ventricular myocardium enables more harmonious alignment towards adequate loading conditions of the left ventricle.

From our point of view 2 exceptions in using fenestrated ASD-closure have been left. First is in the context of staged left ventricular recruitment after single-ventricle palliation. In patients who were taken from univentricular to biventricular pathway, Emani and colleagues used a 4 mm fenestrated patch in order to increase left ventricular end-diastolic volume. Surgical restriction of the atrial septum was the only predictor of a successful biventricular repair [19]. Second, in selected patients with right heart obstructive lesions like pulmonary atresia, a fenestration enabling “rescue” right-to-left shunting in situations with elevated pulmonary resistance can be helpful to ensure a sustained systemic ventricular output Similarly in patients with Fontan-circulation where a fenestration between the Fontan-tunnel and systemic atrium is commonly performed “blue output is better than no output” [20,21]. Mignosa described the case of a patient who underwent arterial switch operation with closure of an aortopulmonary window. A left-open IAC leaving a 4 mm fenestration was established enabling sufficient cardiac output by right-to-left shunting on atrial level in case of threating postoperative pulmonary hypertensive crisis [21].

LVOTO is often caused by posterior malalignement of the conal septum, which is a common finding in lesions with aortic arch hypoplasia or interruption, and is distinctive for the degree of malformation. The minimal diameter of the left ventricular outflow tract enabling a successful repair is still a matter of debate and was not subject of this article. An association between LVOTO and the need for subsequent procedures, or the impact of LVOTO on survival has been described in The Congenital Heart Surgeons Society study (CHSS) after evaluation of 472 neonates with interrupted aortic arch. Interestingly, a correlation between the necessity to patch a hypoplastic arch and later reoperation at the left ventricular outflow tract was found, confirming a pathophysiological or morphological association between narrowed left sided structures [5]. Another morphological characteristic influencing blood flow and growth of cardiac structures, which correlated with re-intervention at the left ventricular outflow tract, was mentioned by Morales and colleagues in the context of type B arch interruption repair. The authors suggested that patients with anomalous take off of the right subclavian artery have even less blood flow through the left ventricle than other patients with interrupted aortic arch, because the flow is only supplying the right and left carotid arteries. This, in addition to the decreased flow through the left ventricle secondary to intra-cardiac shunting and ductal patency may further increase the severity of left ventricular outflow tract underdevelopment [6].

The retrospective nature of this study imposed several inherent limitations. The study cohort was small and included a heterogeneous group of selected patients with aortic arch obstruction. The follow-up period was relatively short and all patients will need continuing assessment, and their long-term outcomes remain uncertain. Echocardiographic measurements did not include left ventricular volume or indexed valve size measurements. Other studies have pointed out exceptions of a successful biventricular repair represented by anatomic variants like moderate/large ventricular septal defect despite a small left ventricle, uni-commissural aortic valve, lower mitral valve dimension z-score [22] or lesions which result in decreased diastolic compliance, i.e. aortic stenosis and endocardial fibroelastosis, [23,24] which commonly occur together. Unfortunately mitral valve Z-scores and endocardial fibroelastosis indicating chronic sub-endocardial ischemia were not assessed in this study.

## Conclusion

In conclusion, a left open IAC following neonatal primary repair for aortic arch obstruction increases the probability of re-intervention at the left heart aorta complex. It seems in the long-term, that a continuous steal in cardiac output by an open IAC impairs growth of left heart cardiac structures.

## Abbreviations

ASD: Atrial septal defect
AVR: Aortic valve repair (commissurotomy)
CI: Confidence interval
CPB: Cardiopulmonary bypass
DHCA: Deep hypothermic circulatory arrest
HAA: Hypoplastic aortic arch
IAA: Interrupted aortic arch
IAC: Inter-atrial communication
IVS: Intact ventricular septum
LCO: Low cardiac output
LVOTO: Left ventricular outflow tract obstruction
OD: Odds ratio
PFO: Persistent foramen ovale
Re-CoA: Aortic re-coarctation
SD: Standard deviation
VSD: Ventricular septal defect
X-clamp: Aortic cross-clamp
